# Transgene integration causes RARB downregulation in homozygous Tg4–42 mice

**DOI:** 10.1038/s41598-020-63512-8

**Published:** 2020-04-14

**Authors:** Barbara Hinteregger, Tina Loeffler, Stefanie Flunkert, Joerg Neddens, Ruth Birner-Gruenberger, Thomas A. Bayer, Tobias Madl, Birgit Hutter-Paier

**Affiliations:** 1 QPS Austria GmbH, Parkring 12, 8074 Grambach, Austria; 2 Gottfried Schatz Research Center (for Cell Signaling, Metabolism and Aging) Division of Molecular Biology and Biochemistry, Medical University of Graz, Graz, Austria; 3 Diagnostic and Research Institute of Pathology & Omics Center Graz, Medical University of Graz, Graz, Austria; 4 BioTechMed-Graz, Graz, Austria; 5 Vienna University of Technology, Institute of Chemical Technologies and Analytics, Vienna, Austria; 6 Department of Psychiatry and Psychotherapy, Division of Molecular Psychiatry, University Medical Center Göttingen (UMG), Göttingen, Germany

**Keywords:** Alzheimer's disease, Neurodegeneration

## Abstract

Alzheimer’s disease can be modelled by different transgenic mouse strains. To gain deeper insight into disease model mechanisms, the previously described Tg4–42 mouse was analysed for transgene integration. On RNA/DNA level the transgene integration resulted in more than 20 copy numbers and further caused a deletion of exon 2 of the retinoic acid receptor beta. These findings were also confirmed on protein level with highly decreased retinoic acid receptor beta protein levels in homozygous Tg4–42 mice and may have an impact on the previously described phenotype of homozygous Tg4–42 mice to be solely dependent on amyloid-ß 4–42 expression. Since hemizygous mice show no changes in RARB protein levels it can be concluded that the previously described phenotype of these mice should not be affected by the retinoic acid receptor beta gene knockout. In order to fully understand the results of transgenesis, it is extremely advisable to determine the genome integration site and the basic structure of the inserted transgenes. This can be carried out for instance by next-generation sequencing techniques. Our results thus suggest that a detailed characterization of new disease models using the latest genomics technologies prior to functional studies could be a valuable tool to avoid an unexpected genetic influence on the animals’ phenotype that is not only based on the inserted transgene. This would also significantly improve the selection of mouse models that are best suited for therapeutic development and basic research.

## Introduction

Alzheimer’s disease (AD) is the most common neurodegenerative disorder and mainly characterized by progressive neurodegeneration as well as extracellular accumulation of amyloid-ß protein (Aß) that is surrounded by dystrophic neurites and neurofibrillary tangles^1^. To gain deeper insight into the underlying disease mechanisms the use of AD animal models in research is highly relevant and thus far without alternative^2,3^. While cognitive deficits, amyloid deposits or neurofibrillary tangles and inflammation are observable in many AD mouse models, neurodegenerative pathology as characterized by e.g. hippocampal neuron loss is only observed in a few models^4–9^. For this reason, we further characterized the previously described Tg4–42 mouse model, as it develops neuronal loss and therefore represents a promising model for the development of new drugs or drug efficacy studies^10^. Homozygous Tg4–42 mice are characterized by an age depended, massive CA1 pyramidal neuronal loss in the hippocampus. Furthermore, synaptophysin staining in the CA3 region of the hippocampus demonstrates a modified synapse pattern in these mice, which might be caused by a possible disturbance of the neuronal network. Homozygous Tg4–42 mice exhibit no significant target quadrant preferences in the Morris water maze behaviour test, which indicates severe impairments in spatial memory. According to AD pathologies these mice show age-dependent impaired glucose metabolism in all cortical areas which were analysed by 18F-FDG-PET^11^. In contrast hemizygous Tg4–42 mice still demonstrate a significant preference for the target quadrant like wild type mice in the Morris water maze behavioural test at the same tested age, indicating no or only weak impairments in spatial memory. Moreover, also synaptic chances were less pronounced in hemizygous compared to homozygous mice^12^. These analyses reveal that the gene-dosage influences the phenotype of these mice. Homozygous mice develop enhanced neuropathological features and neuronal loss compared to hemizygous animals at the same age^13^.

Two different studies exhibited a rescue of this phenotype by therapeutic interventions. Homo- and hemizygous Tg4–42 mice that were allowed to exercise in enriched housing conditions revealed improved performance on the balance beam, rotarod and rescued performance in the Morris water maze test. Additionally, this increased physical activity caused decreased neuron loss which resulted in a higher number of CA1 pyramidal cells^7^. A weekly treatment with the full-length or Fab fragment of a monoclonal antibody directed against Aß4–42 also showed the ability to completely rescue spatial reference memory deficits and significantly stabilized neuron numbers in 6 month old homozygous Tg4–42 mice^10^.

To further characterize the genetic features of Tg4–42 mice, whole genome sequencing was performed to determine the integration site(s) of the transgene and estimate the transgene copy number as well as assess the presence of structural variants surrounding the transgene integration site and the transgene sequence itself. Decreased expression and reduced protein levels of the retinoic acid receptor beta in brain tissue of homozygous Tg4–42 mice was found, questioning the cause of the previously described phenotype in homozygous Tg4–42 mice to be solely dependent on Aß4–42 expression.

## Results

### Gene expression analyses

**Figure 1:**
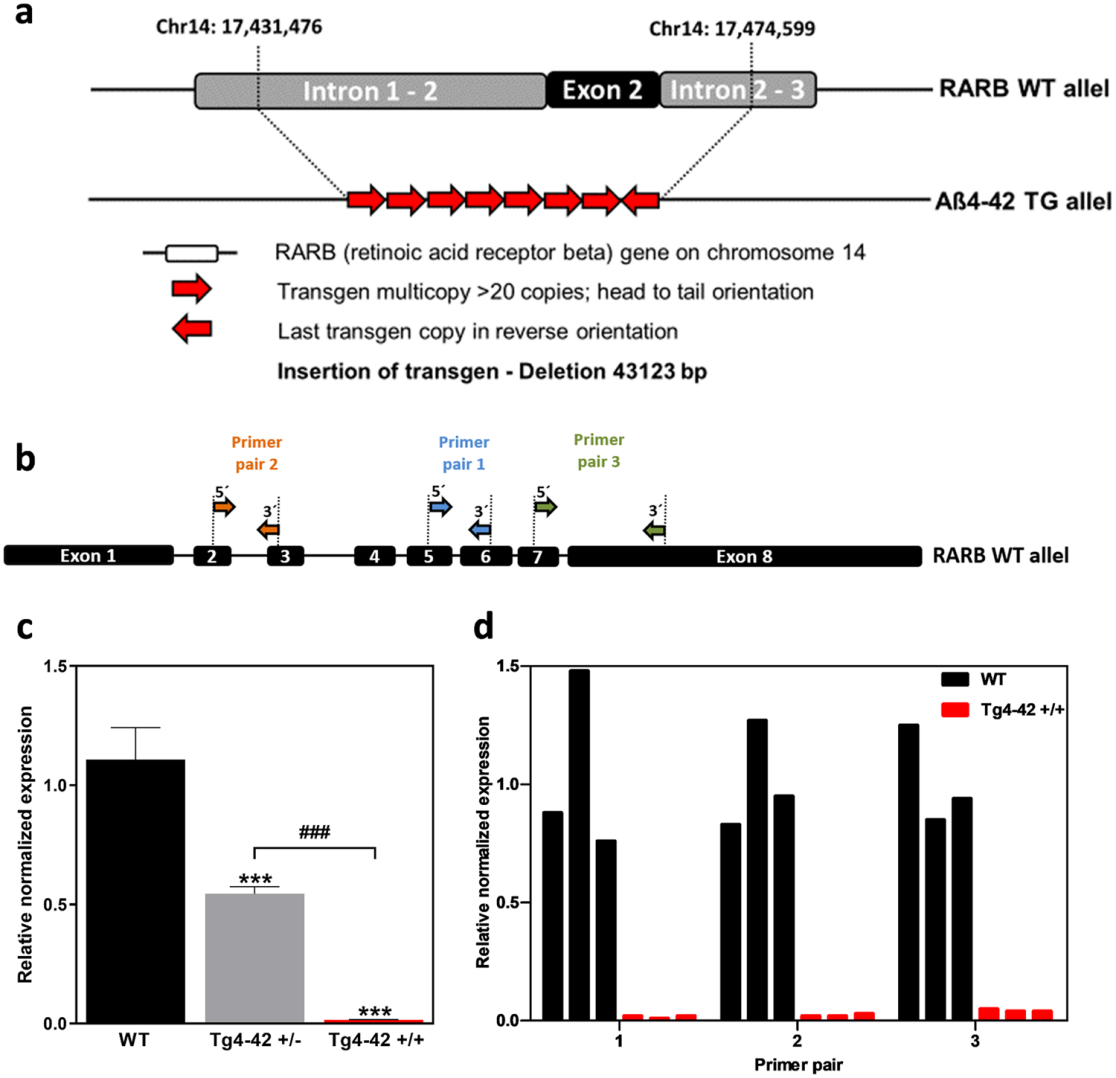
Gene expression analyses of Tg4–42 + /+ mice. (**a**) Whole genome sequencing showing the transgene integration site and the deletion of RARB exon 2 on the mouse genome. (**b**) Binding sites of three different primer pairs used to detect the expression levels of the retinoic acid receptor beta. (**c**) Gene expression levels measured with conventional qPCR by primer pair 1 in three wild type (WT), three hemizygous (Tg4–42 + /−) and three homozygous Tg4–42 (+/+) striatal samples after cDNA synthesis. HPRT was used as a housekeeping gene to normalize expression levels of the RARB gene. One-way ANOVA followed by Tukey’s multiple comparisons test (n = 3 per group. Mean + SEM. F (2, 6) = 146.6, R^2^ = 0.9794. Significances are labelled as followed: ***p < 0.001 against WT and ^###^ as indicated). (**d**) qPCR results of three wild type (WT) and three homozygous Tg4–42 (+/+) striatal samples using primer pair 1, 2 and 3. Mean Cq values of RARB were normalized with mean Cq values of HPRT.

Targeted Locus Amplification of the Tg4–42 + /+ mouse model showed that the Aβ4–42 transgene is inserted in more than 20 copies of head to tail orientation at position 17,431,476–17,651,381 (mouse genome assembly mm9) on chromosome 14 (see Supplementary Information). Only the last transgene copy is in reverse orientation. Furthermore, exon 2 of the retinoic acid receptor beta (RARB) gene was deleted in Tg4–42 mice (Fig. 1a).

To analyse how this genetic deletion influences RARB RNA expression levels and if expression of whole mRNA is affected, primer pair 1 which binds between exon 5 and 6 was designed. Quantitative PCR using primer pair 1 showed expression levels that were almost reduced to 50% in hemizygous Tg4–42 + /− and almost immeasurable in homozygous Tg4–42 + /+ animals (Fig. 1c). To validate that the same amount of cDNA was used in all samples, HPRT was used as housekeeping gene.

To confirm these results, two further primer pairs were used binding from exon 2 to 3 and from exon 7 to 8 and thus outside of the deleted gene region of RARB (Fig. 1b).

qPCR was further performed using cDNA of three different homozygous Tg4–42 + /+ and three wild type (WT) striatal samples. Results show that all three primer pairs showed a significant knockdown of RARB RNA levels in all Tg4–42 + /+ animals (Fig. 1d).

### Protein analysis by Western blotting, Mass spectrometry and Simple Western blot system

To confirm the gene expression results on protein level Western blot analysis was performed. Many different approaches like two different protein extraction methods, different protein concentrations, three different primary antibodies (ab53161, Abcam; orb11327, Biorbyt; sc-514585, Santa Cruz Biotechnology; dilution 1:500), different incubation settings and different gel detection methods were used but did not show satisfactory results because of the non-specificity or low sensitivity of used antibodies (data not shown).

Furthermore, protein analysis with Mass spectrometry was carried out in an untargeted as well as targeted approach but the concentration of RARB protein in WT animals was under the limit of detection.

**Figure 2:**
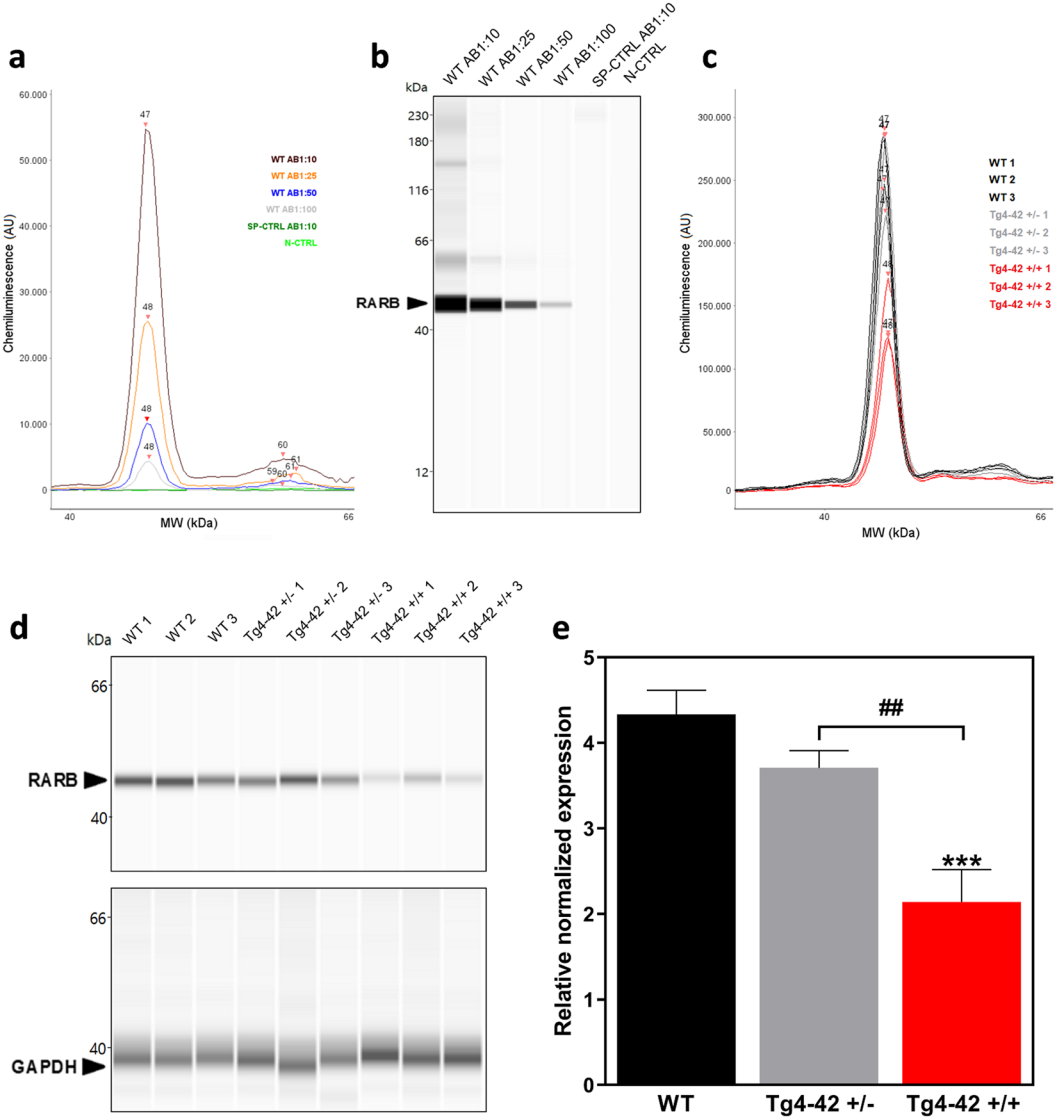
Simple Western protein analysis of the retinoic acid receptor beta in wild type (WT) and Tg4–42 + /+ mice. (**a, b**) Different antibody dilutions of a monoclonal retinoic acid receptor beta 2 antibody were tested with the Simple Western blot system to determine its binding capacity and optimal antibody concentration. A specificity control (SP-CTRL) and a negative control (N-CTRL) were used to detect unspecific binding of the column and cross reactions, respectively. (**c, d**) After protein extraction, striatal protein samples of three different WT, Tg4–42 + /− and Tg4–42 + /+ mice were measured by the Simple Western system. GAPDH was used as loading control. (**e**) Quantification of RARB protein of three wild type (WT), three hemizygous (Tg4–42 + /−) and three homozygous Tg4–42 (+/+) striatal samples as shown in (**c**, **d**) normalized to GAPDH. One-way ANOVA followed by Tukey’s multiple comparisons test (n = 3 per group. Mean + SEM. F (2, 6) = 43.4, R^2^ = 0.9354, Significances are labelled as followed: ***p < 0.001 against WT and ^##^p < 0.01 as indicated).

As the traditional Western blot as well as the mass spectrometry analysis did not show any useful result on protein level, Simple Western blot analysis was performed. Therefore, a monoclonal retinoic acid receptor beta 2 antibody (sc-514585, Santa Cruz Biotechnology) already unsuccessfully used for classical Western blotting was tested in different concentrations. The antibody showed a specific signal at 48 kDa in WT animals which decreased at higher antibody dilutions. Moreover, the specificity control (SP-CTRL) without a biological sample, to evaluate if the primary antibody sticks to capillary wall material and a negative control (N-CTRL) without the primary antibody to check for cross-reactivity, showed no unspecific signal (Fig. 2a,b). For further experiments the lowest tested antibody dilution (1:10) was used. In this study we focused on the analysis of homozygous Tg4–42 mice compared to wild type animals to clearly visualize differences in retinoic acid receptor beta protein levels caused by RARB gene knockout. Nevertheless, to confirm the qPCR data total protein was extracted from three homozygous Tg4–42 + /+, three heterozygous Tg4–42 + /− and three wild type striatal samples and measured by the Simple Western blot system. The analysis revealed a significant knockdown of retinoic acid receptor beta protein in Tg4–42 + /+ mice compared to wild type and also Tg4–42 + /− mice and therefore confirmed the qPCR results. GAPDH was used as loading control (Fig. 2c,e).

## Discussion

In this study we used the Tg4–42 mouse model which was previously introduced and described to express N-truncated amyloid-β 4–42. Homo- and hemizygous Tg4–42 animals develop a typical AD phenotype, including neuronal loss and spatial learning deficits albeit without plaque formation^7,12,14^.

Using qPCR, we have shown the transcriptional impact of transgene integration in homozygous Tg4–42 mice. Whole genome sequencing located the integration site of the transgene on chromosome 14, which caused a deletion of exon 2 of the retinoic acid receptor beta. TLA combined with next generation sequencing is best suited for a robust detection of integration sites or transgene sequence variants^15,16^. For further validation of the genomic integration site, Sanger sequencing could be performed, although Baudhuin and colleagues question the necessity of this confirmation^17^. To clarify the impact of transgene insertion on the protein level, we used the Simple Western blot system. This novel capillary electrophoresis-based Western blot technology requires much smaller amounts of tissue than traditional Western blot which is important if limited sample amounts are available. Furthermore, the higher sensitivity as well as reproducibility and excellent quantification options by analysis of the *Area Under the Curve* are big advantages which led to the decision to use the capillary Western approach in our study^18,19^. The Simple Western blot system analyses resulted in a highly decreased RARB protein level in homozygous Tg4–42 mice. Thus, the integration of the Aβ4–42 transgene appears to substantially disrupt RARB gene regulation, resulting in a knockdown of this gene and in turn downregulation of RARB protein. The described phenotype of the homozygous Tg4–42 mouse model could therefore be caused by the Aβ4–42 transgene or by RARB knockout^20^.

A generated RARB null mutant mouse model described in the literature with a disruption of all isoforms of RARB shows a very similar phenotype compared to homozygous Tg4–42 mice^21,22^. The retinoic acid receptor beta deficiency eliminates hippocampal CA1 long-term potentiation and causes severely impaired spatial learning in the Morris water maze behavioural test, like it is described in homozygous Tg4–42 animals. In contrast, hemizygous Tg4–42 animals still show target zone preferences, which might also be attributed to unaltered RARB protein levels. These generated RARB knockout mice are viable, fertile and display no obvious defects^12,23^. Nevertheless, it is described that a loss of both wild type alleles of the RARB gene leads to a cell deficit on the ganglion cell population, which of course can influence the phenotype^24^. Otherwise, therapeutic intervention studies with a monoclonal antibody or with enriched housing conditions were able to reduce hippocampal neuron loss and showed a rescue of spatial reference memory deficits in homozygous Tg4–42 mice. These data would rather indicate that the observed phenotype of homozygous Tg4–42 mice is caused by the Aβ4–42 transgene^7,10^. Another conclusion from the current work is that it should be obligatory to analyse a possible treatment effect in more than one Alzheimer’s disease mouse model. Since hemizygous mice showed no changes in RARB protein levels, the previously described phenotype of these mice should not be affected by the retinoic acid receptor beta gene knockout. Our results demonstrate that it is extremely advisable and helpful to determine the genomic integration site and the basic structure of the inserted transgenes for instance by next-generation sequencing techniques to be able to fully understand the effects of transgenesis. In order to avoid an unexpected genetic impact on the animals’ phenotype, which is not only based on the inserted transgene a detailed characterization of new disease models using the latest genomics technologies is of enormous importance. This would also significantly improve the selection of mouse models that are best suited for basic research and therapeutic development.

## Conclusion

In conclusion, our data provide detailed information about the integration site of the Aβ4–42 transgene and additionally show decreased expression and reduced protein levels of the retinoic acid receptor beta in brain tissue of homozygous Tg4–42 mice. These results question the cause of the previously described phenotype of homozygous Tg4–42 mice to be solely dependent on Aß4–42 expression. Further studies need to address this issue in detail.

## Material and Methods

### Animals

Male homozygous (Tg4–42 + /+), hemizygous (Tg4–42 + /−) and non-transgenic wild type (WT) control mice were bred and housed in individually ventilated cages in a controlled environment on a 12/12-h light/dark cycle (lights on from 6 a.m.–6 p.m.), with humidity between 40 to 70%, temperature of approximately 21 °C and food and water *ad libitum* in an AAALAC accredited animal facility. All animal studies adhered to the Austrian law for the care and use of laboratory animals.

Mice were deeply anesthetized by i.p. injection of 600 mg/kg pentobarbital at the age of 6 months and transcardially perfused with physiological (0.9%) saline. Thereafter, brains were removed, hemisected and subdivided into different brain areas, immediately frozen on dry ice and stored at −80 °C.

### Targeted locus amplification and sequence alignment

Targeted Locus Amplification (TLA) of Tg4–42 mice was performed by Cergentis (Utrecht, Netherlands) as previously descried in de Vree *et al*.^15^. PCR products were purified and library prepped using the Illumina NexteraXT protocol and sequenced on an Illumina Miseq sequencer. Reads were mapped using BWA-SW, which is a Smith-Waterman alignment tool. This allows partial mapping which is optimally suited for identifying break spanning reads. For mapping the mouse genome, version mm9 (MGSCv37) was used by this company.

### mRNA analysis (RNA extraction, cDNA synthesis and quantitative PCR)

Total striatal RNA was extracted using peqGOLD TriFast Kit (VWR Life Science, Erlangen, Germany). 1 µg of total RNA was reverse-transcribed into single stranded cDNA using iScript gDNA Clear cDNA Synthesis Kit (Bio-Rad Laboratories, Hercules, USA). Quantitative PCR (qPCR) was performed with Bio-Rad CFX Connect thermo cycler using Takyon No Rox SYBR MasterMix dTTP Blue (Eurogentec, Seraing, Belgium) with the primers (Microsynth, Balgach, Switzerland) listed in Table 1. The ∆∆Cq method was applied to calculate the relative fold-change in gene expression after normalization to HPRT (Hypoxanthin-Guanin-Phosphoribosyl-Transferase).

**Table 1:** List of primers.

Gene	Primer Sequence
RARB primer pair 1	F: GCCTCTGGGACAAATTCAGT R: GTCAGTCAGAGGACCGAAGC
RARB primer pair 2	F: CTGCTTCGTTTGCCAGGACA R: GGAAAAAGCCCTTGCACCC
RARB primer pair 3	F: CGAAAGGTGCCGAACGTGTA R: TGAACTTGGGGTCAAGGGTT
HPRT	QuantiTect Primer Assay HPRT_1 (Quiagen, Germany)

RARB: Retinoic acid receptor beta; F: Forward primer sequence; R: Reverse primer sequence; HPRT: Hypoxanthin-Guanin-Phosphoribosyl-Transferase.

All PCR amplifications were performed in a total volume of 20 µl, containing 2 µl of cDNA, 10 µl of 2x Takyon SYBR mix, 1 µM of each primer and 6 µl of aqua bidest. The amplification protocol consisted of an initial denaturation at 95 °C for 3 min, followed by 39 cycles of denaturation at 95 °C for 0.2 min, annealing at 60 °C for 0.5 min and extension at 72 °C for 0.5 min.

### Protein sample preparation

Mouse striatal samples were weighted and lysed in 15 volumes RIPA buffer containing protease- and phosphatase-inhibitor and sonicated with the Ultra-Turrax for 30 s. Afterwards tissue homogenates were centrifuged at 20,000 × g at 4 °C for 10 min to remove insoluble material. The protein concentration of tissue homogenates were determined using Pierce BCA protein assay kit (Thermo Scientific, USA). For capillary simple Western blotting homogenates were diluted to 0.65 mg protein/ml sample, using sample buffer.

### Simple Western blot system

Samples were prepared and analysed according to the manufacturer’s instructions (Protein Simple, San Jose, USA). Briefly, four volumes of sample were mixed with one volume of fluorescent 5xMaster Mix (Protein Simple) and denatured at 95 °C for 5 min. Primary antibodies against retinoic acid receptor beta 2 (sc-514585; Santa Cruz Biotechnology, Heidelberg, Germany) and Glycerinaldehyd-3-phosphat-Dehydrogenase (GAPDH; G9545; Sigma-Aldrich, St. Louis, USA) were diluted 1:10 (RARB) and 1:100 (GAPDH) in antibody diluent 2. The samples, biotinylated ladder, primary antibodies, secondary antibodies, chemiluminescent substrate, Stacking Matrix 2 and the Separation Matrix 2 (12–230 kDa; supplied by the manufacturer) were dispensed into the assay plate. After adding Simple Western assay buffers subsequent separation, immunodetection and analysis steps were performed automatically and digitally by the Simple Western blot System.

Compass for SW software (Protein Simple version 4.0.0) was used to visualize Simple Western lanes digitally, automatically analyse signal peaks and calculate the area under the curve of the peak of interest.

### Statistical analysis

All statistical analyses and graphs were performed using GraphPad Prism 7.0 software (GraphPad Software, San Diego, USA).

### Compliance with ethical standards

All experiments including animal tissue were performed in accordance with the Austrian guidelines for the care and use of laboratory animals (Tierversuchsgesetz 2012-TVG 2012, BGBl. I Nr. 114/2012). Animal housing and euthanasia were approved by the Styrian government (Amt der Steiermärkischen Landesregierung, Abteilung 13 – Umwelt und Raumordnung Austria; ABT13-78Jo115/2013-2016; ABT13-78Jo-118/2013-13).

## Supplementary information

Supplementary information.

## Data Availability

All data generated or analysed during this study are included in this manuscript.

## References

[1] Alzheimer’s disease facts and figures. *Alzheimer’s & dementia: the journal of the Alzheimer’s Association* **8**, 131–168, 10.1016/j.jalz.2012.02.001 (2012). 10.1016/j.jalz.2012.02.001 22404854

[2] Alexandru A (2011). Selective hippocampal neurodegeneration in transgenic mice expressing small amounts of truncated Abeta is induced by pyroglutamate-Abeta formation. The Journal of neuroscience: the official journal of the Society for Neuroscience.

[3] Dunkelmann T (2018). Comprehensive Characterization of the Pyroglutamate Amyloid-β Induced Motor Neurodegenerative Phenotype of TBA2.1 Mice. J Alzheimers Dis.

[4] Hall AM, Roberson ED (2012). Mouse models of Alzheimer’s disease. Brain research bulletin.

[5] LaFerla, F. M. & Green, K. N. Animal models of Alzheimer disease. *Cold Spring Harbor perspectives in medicine* **2**, 10.1101/cshperspect.a006320 (2012). 10.1101/cshperspect.a006320 PMC3543097 23002015

[6] Wirths, O. & Bayer, T. A. Neuron loss in transgenic mouse models of Alzheimer’s disease. *Int J Alzheimers Dis* 2010, 10.4061/2010/723782 (2010). 10.4061/2010/723782 PMC2943100 20871861

[7] Huttenrauch M (2016). Physical activity delays hippocampal neurodegeneration and rescues memory deficits in an Alzheimer disease mouse model. Translational psychiatry.

[8] Elder GA, Gama Sosa MA, De Gasperi R (2010). Transgenic mouse models of Alzheimer’s disease. The Mount Sinai journal of medicine, New York.

[9] Mariani MM (2017). Neuronally-directed effects of RXR activation in a mouse model of Alzheimer’s disease. Scientific reports.

[10] Antonios G (2015). Alzheimer therapy with an antibody against N-terminal Abeta 4-X and pyroglutamate Abeta 3-X. Scientific reports.

[11] Bouter C (2019). (18)F-FDG-PET Detects Drastic Changes in Brain Metabolism in the Tg4-42 Model of Alzheimer’s Disease. Frontiers in aging neuroscience.

[12] Bouter Y (2013). N-truncated amyloid beta (Abeta) 4-42 forms stable aggregates and induces acute and long-lasting behavioral deficits. Acta neuropathologica.

[13] Lopez-Noguerola JS (2018). Synergistic Effect on Neurodegeneration by N-Truncated Aβ and Pyroglutamate Aβ in a Mouse Model of Alzheimer’s Disease. Frontiers in aging neuroscience.

[14] Bouter Y (2014). Deciphering the molecular profile of plaques, memory decline and neuron loss in two mouse models for Alzheimer’s disease by deep sequencing. Frontiers in aging neuroscience.

[15] de Vree PJ (2014). Targeted sequencing by proximity ligation for comprehensive variant detection and local haplotyping. Nat. Biotechnol..

[16] Tosh JL (2018). The integration site of the APP transgene in the J20 mouse model of Alzheimer’s disease. Wellcome Open Res..

[17] Baudhuin LM (2015). Confirming Variants in Next-Generation Sequencing Panel Testing by Sanger Sequencing. J. Mol. Diagn..

[18] Nguyen U, Squaglia N, Boge A, Fung PA (2011). The Simple Western™: a gel-free, blot-free, hands-free Western blotting reinvention. Nature Methods.

[19] Lu, J., Allred, C. C. & Jensen, M. D. Human adipose tissue protein analyses using capillary western blot technology. *Nutr. Diabetes.* **7**, e287, 10.1038/nutd.2017.35 (2017). 10.1038/s41387-018-0030-4 PMC5916899 29695704

[20] Woychik RP, Alagramam K (1998). Insertional mutagenesis in transgenic mice generated by the pronuclear microinjection procedure. The International journal of developmental biology.

[21] Luo J, Pasceri P, Conlon RA, Rossant J, Giguere V (1995). Mice lacking all isoforms of retinoic acid receptor beta develop normally and are susceptible to the teratogenic effects of retinoic acid. Mechanisms of development.

[22] Nomoto M (2012). Dysfunction of the RAR/RXR signaling pathway in the forebrain impairs hippocampal memory and synaptic plasticity. Molecular brain.

[23] Chiang MY (1998). An essential role for retinoid receptors RARbeta and RXRgamma in long-term potentiation and depression. Neuron.

[24] Zhou, G., Strom, R. C., Giguere, V. & Williams, R. W. Modulation of retinal cell populations and eye size in retinoic acid receptor knockout mice. *Mol. Vis.* **7**, 253–260 (2001). 11723443

