# Supplementary material for: Transgene integration causes RARB downregulation in homozygous Tg4–42 mice
Source: Sci Rep. 2020 Apr 14;10:6377. doi: 10.1038/s41598-020-63512-8 (PMC7156671; doi:10.1038/s41598-020-63512-8)
Supplement: Supplementary file 1 — Supplementary information. [file 41598_2020_63512_MOESM1_ESM.pdf]

Supplementary Information

Transgene integration causes RARB downregulation in homozygous Tg4-42 mice

Barbara Hinteregger, Tina Loeffler, Stefanie Flunkert, Joerg Neddens, Ruth Birner-Gruenberger, Thomas A. Bayer, Tobias Madl, Birgit Hutter-Paier

Complete Gene Sequencing Report Cergentis (Utrecht, Netherlands)

Goal:

In this study a transgenic mouse Tg4-42 (TBA83) line was analyzed.

The aim of this experiment was to determine:

1. the integration site(s) of the transgene (TG)

2 assess the presence of structural variants surrounding the transgene integration site

3. assess the transgene sequence itself

4. estimate the TG copy number

Summary of the Results:

Name	Integration site(s)	Estimated Copy nr	SV
TBA83; Mouse spleen	mChr14:17431476-17474599	>20x	yes

Experiment:

TLA sample prep:

Isolated mouse spleen sample from the transgenic line was used for TLA sample prep.

TLA PCR:

Two primer pairs (set 6-7) were designed for the mouse TG.

Primers used in the analysis:

#	Name	position	Sequence
6	THR-Abeta4-42-F	TG:4350	CTGCCTTAGATTCCTGGATC
	THR-Abeta4-42-R	TG:4121	GACTTGGATTCTGTGGTGG
7	Thy-F	TG:5839	CAAATCAATGCGCATTCTCT
	Thy-R	TG:5578	TTTCTTTCCTCGGTGAGATG

The primer sets were used in individual TLA amplifications. PCR products were purified and library prepped using the Illumina NexteraXT protocol and sequenced on an Illumina Miseq sequencer. Reads were mapped using BWA-SW, which is a Smith-Waterman alignment tool. This allows partial mapping which is optimally suited for identifying break spanning reads. For mapping the mouse genome version mm9 was used.

Results - Integration site:

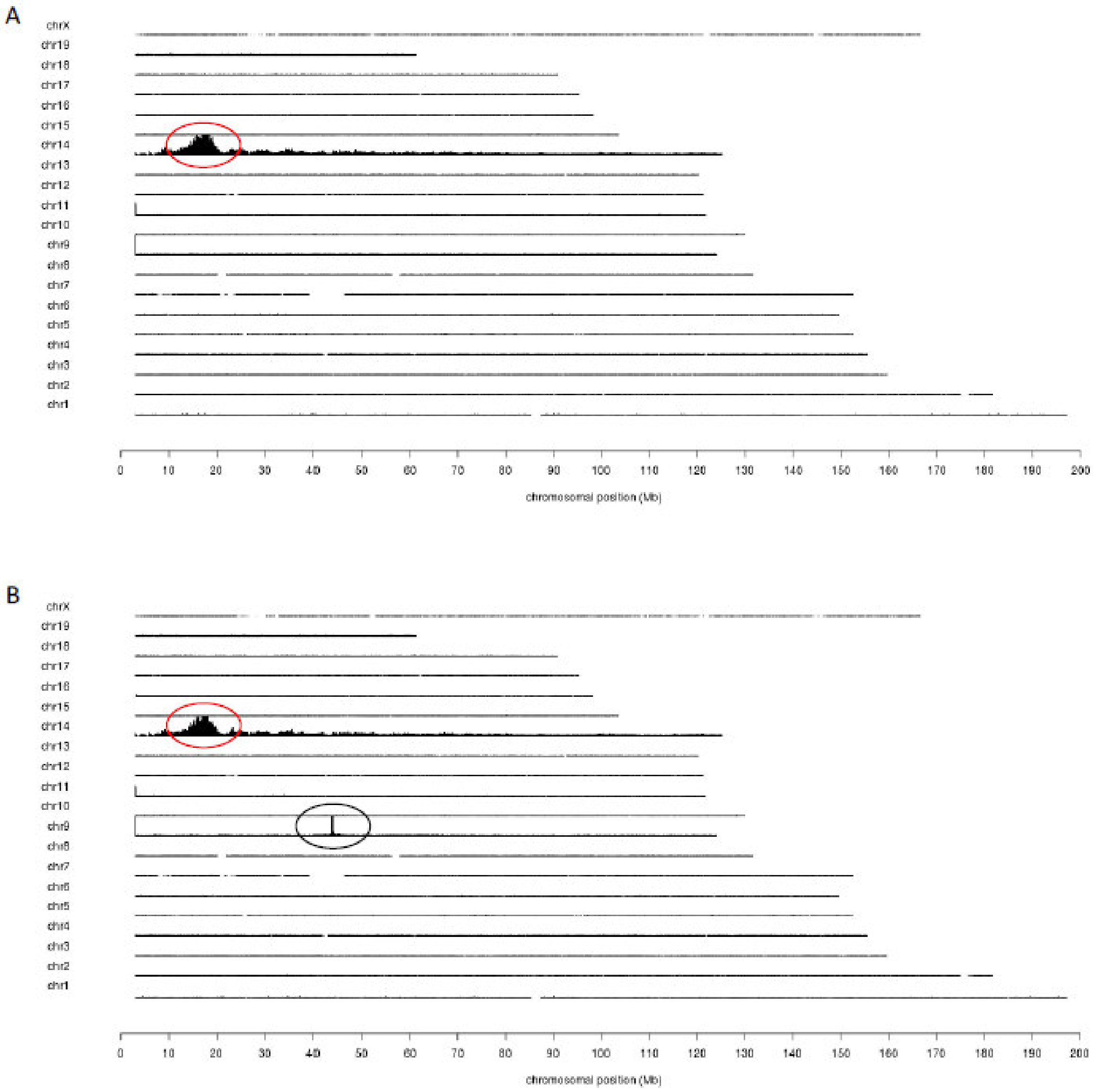

TLA sequence coverage across the mouse genome using primer set 6 (panel A) and set 7 (panel B). The different chromosomes are indicated on the y-axis, the chromosomal position on the x-axis. Similar results were obtained for both primer sets except using set 7 an additional coverage peak is found on the mouse Thy1 gene as expected (black circle). Encircled in red is the region containing the transgene integration site.

Using TLA highest coverage is observed on the sequences directly surrounding the location of the primer set. Here high coverage is observed on chr14 indicating this is the chromosome where the TG has integrated. Within chr14 highest coverage is observed in the region 15-25MB. Within this region the following fusion reads were identified marking the exact TG integration.

5’ fusion read: mChr14:17431476 (forw) fused to TG:4345 (forw)

..GTGCTCCTGCCTGCAGTCAGGATGAACCCCTTCTAAGATGATCTACAGCATTGCC
CCCACTCACTGGTGCTGCCTTAGATTCCTGGATCACAAAACGCTTCCGACATGAC
TCAGGATATGAAGTTCATCATCAAAAATTGGTGTTCTTTG..

3’ fusion read: TG:6724 (rev) fused to mChr14:17474599 (forw)

..GCCTCAGAGAGCACCCGCATCTCCCTAAGAACTGAGAGTGTGGTCACGGAGATG
TCCAGGGA
AGAGCACCTAGTGTGACAGAGACCACATCATATGACTCTGTAGGCAAGTCTGCTG
CAGATCGCCTTTGGCACCCTTCAACTGCCGACAA..

As a result the TG was found to be integrated at mChr14:17431476-17474599 deleting all sequences in between.

Results - Transgene Sequence:

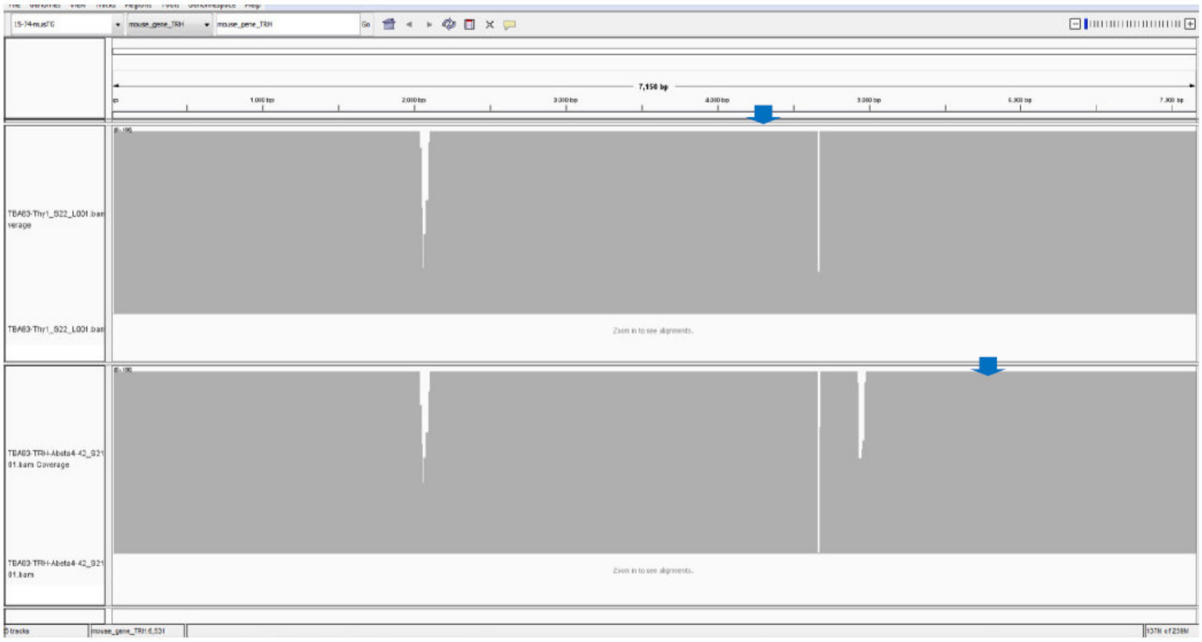

IGV screenshot of the TLA sequence coverage across the TG. Sequence coverage (in grey) generated with the different primer sets are depicted in the different panels as indicated. The positions of the primer sets on the TG sequence are indicated with the blue arrows. Y-axis is limited to 100x. Good coverage is observed across the TG except for TG:4642-4689 due to the high GC content of that region. From these data the following sequence variation was identified that was found with a frequency of minimal 10% using minimal 1 primer set. Complete tables are available from Cergentis upon request.

SNVs			TBA83-Thy1	TBA83-Thy1	TBA83-TRH-Abeta4-42	TBA83-TRH-Abeta4-42
pos	ref	alt	cov	SNP_%	cov	SNP_%
3692	C	T	996	17	44080	21
3744	C	T	753	95	37852	99
3761	G	T	835	94	42394	99
3762	C	T	837	93	42420	98
4961	T	G	12136	76	42	100
5136	G	A	47534	14	213	14
5298	C	T	71479	93	329	100
5996	A	G	50674	100	512	100
6645	G	A	3474	4	9773	21

INDELs			TBA83-Thy1	TBA83-Thy1	TBA83-TRH-Abeta4-42	TBA83-TRH-Abeta4-42
pos	ref	alt	cov	SNP_%	cov	SNP_%
4497	A	-6AGATGT	688	51	70302	64
4505	C	-7AAGTAAG	357	99	45594	99
4941	A	-2GG	9920	31	40	35
4941	A	-3GGG	9920	8	40	10
5925	A	-1G	60539	92	476	86
5927	A	+2CC	56287	99	417	97
5953	T	-1G	78485	98	615	98
5987	A	+1G	74918	67	656	77
5989	C	-1G	50752	99	512	99
5991	C	-2CT	50750	99	512	99
6003	A	-1G	50800	100	512	100

Results - Structural Variation:

The presence of fusion reads within the TG sequence allows for the detection of (potential) structural variation within the TG. Multiple fusion reads were identified in the TG sequence which are reported in the table below.

#seq1	pos1	ori1	seq2	pos2	ori2
mouse_gene_TRH	2350	+	mouse_gene_TRH	3286	+
mouse_gene_TRH	31	+	mouse_gene_TRH	2749	-
mouse_gene_TRH	2390	-	mouse_gene_TRH	3286	+

In addition the following read was found indicative of TG concatemers:

TG:7157 (forw) fused to ‘Unknown’ fused to TG:26 (forw)

GTCCTTATTCTCTCTCTACCTTCAGCCACTTAGTTTCCTACCTTAAGTCCTAGAATT
GATCCTGGCGTAATAGCGAAGAGGCCCGCACCGGGGCTGCAGGAATTCAGAGAC
CGGGAACCAAACTAGCCTTTAAAAAACATAAGTACAGGAG

Results - Copy number estimation:

An exact copy number cannot be determined using TLA. However, an estimation can be made based on the number of integration sites, number of fusion reads and the ratio of coverage on the TG and genome integration site. Here the TG/genome coverage ratio can be determined best, being between 1250 and 3000x on the TG and ~10x and 75x at the integration site on chromosome 14. From this the TG copy number is estimated to be >20x.
